# Focal DETR: Target-Aware Token Design for Transformer-Based Object Detection

**DOI:** 10.3390/s22228686

**Published:** 2022-11-10

**Authors:** Tianming Xie, Zhonghao Zhang, Jing Tian, Lihong Ma

**Affiliations:** 1 School of Electronics & Information Engineering, South China University of Technology, Guangzhou 510640, China; 2 National Research Center for Mobile Ultrasonic Detection, Guangzhou 510640, China; 3 Institute of Systems Science, National University of Singapore, Singapore 119615, Singapore

**Keywords:** object detection, self attention, query-key similarity, vision transformer

## Abstract

In this paper, we propose a novel *target-aware token design* for transformer-based object detection. To tackle the target attribute diffusion challenge of transformer-based object detection, we propose two key components in the new target-aware token design mechanism. Firstly, we propose a *target-aware sampling module*, which forces the sampling patterns to converge inside the target region and obtain its representative encoded features. More specifically, a set of four sampling patterns are designed, including small and large patterns, which focus on the detailed and overall characteristics of a target, respectively, as well as the vertical and horizontal patterns, which handle the object’s directional structures. Secondly, we propose a *target-aware key-value matrix*. This is a unified, learnable, feature-embedding matrix which is directly weighted on the feature map to reduce the interference of non-target regions. With such a new design, we propose a new variant of the transformer-based object-detection model, called *Focal DETR*, which achieves superior performance over the state-of-the-art transformer-based object-detection models on the COCO object-detection benchmark dataset. Experimental results demonstrate that our Focal DETR achieves a 44.7 *AP* in the coco2017 test set, which is 2.7 *AP* and 0.9 *AP* higher than the DETR and deformable DETR using the same training strategy and the same feature-extraction network.

## 1. Introduction

Object detection is important in computer-vision applications such as surveillance, robot vision, automatic driving, and UAV scene analysis. Deep-neural-network-based detection can be divided into three categories according to whether target positioning and target detection are separated: (i) the two-stage algorithms that nominate candidate regions first and then detect the targets; (ii) the end-to-end single-stage algorithms, and (iii) the recently developed transformer-based algorithms.

### 1.1. Two-Stage Object Detection

Among the two-stage detection algorithms, nomination-box methods and location-scoring methods are the most conventional methods. R-CNN [1] is the first CNN-based boxing candidate method including SVM classifiers for all categories. It achieves a higher accuracy than traditional algorithms but fails in computation and memory efficiency. Fast-RCNN [2] algorithms normalize entire images and add a regression-results bounding box to avoid the overlapping of feature extraction. Although its training and testing are fast, the selective nomination boxes lead to inefficient outcomes. Faster-RCNN [3] applies the regional nomination network RPN instead of the search window to improve nomination efficiency and accuracy; its disadvantage lies in the independent target location. R-FCN [4] method adds a location score map to the final full convolution layer to reflect the importance of a location to a target, which is, indeed, a naive attention calculation. These two-stage detectors divide detection tasks into two small networks with simple functions, high detection accuracy, and easy training module, while low speeds of detection and higher computational costs are their weak points.

### 1.2. One-Stage Object Detection

The *You Only Look Once* (YOLO) series treats object-detection tasks as regression and computes the confidence probability of target position and category. It is fast and has a low false-detection rate, but a poor recognition-position accuracy, and a low recall rate. *Single shot multibox detector* (SSD) [5] uses a multi-scale feature map to improve the detection position accuracy in YOLOv1 [6], replaces the last FC layer, and sets an a-priori frame. To improve the optimal scale used in YOLOv2 [7], YOLOv3 [8] uses the *feature pyramid network* (FPN) and improves the hitting of multi-scale targets. YOLOv4 [9] introduces a single-stage anchor box which divides regions through a dense detector. RetinaNet [10] proposes focal loss, focusing on the difficult samples by suppressing the weights of the samples, and uses a classification difficulty loss to control the proportion of positive and negative samples.

### 1.3. Transformer-Based Object Detection

The transformer-based global self-attention detection model *DEtection TRansformer* (DETR) [11] shares the feature key values between the encoder and the decoder as tokens for self-attention modeling, with a randomly initialized object query and matching algorithm. Its query output carries the category and bounding box information by gradually decoding and outputs all detection results at one time in the form of set prediction. Limitations of DETR are (i) high computational complexity due to the global point-to-point calculation; the calculated cost is a quadratic function of the total sample points; and (ii) incoherent interference: since the calculation is point traversing, irrelevant points will interfere, and cause model divergence. Deformable DETR [12] replaces the global self-attention with sparse sampling attention, which applies computation just on reference points and partial sampling points in the neighborhood. The saved computational resources could enable multi-scale attention for high-resolution feature maps without additional FPN [13] modules. Other improvements to DETR concern the way attention is applied and constraints optimization for object queries. For example, conditional DETR [14] decouples the content in cross-attention and spatially matched regions, which can solve the dependence on high-quality embedding. Anchor DETR [15] changes the object query to the encoding of anchor coordinates, with clear location meaning and less optimization difficulty. Efficient DETR [16] uses the Top-*K* generated by dense prediction, scoring encoded features as reference points and object query values, reducing the number of encoder and decoder layers.

D^2^DETR [17] uses an efficient cross-scale attention module without an encoder to generate fused feature maps directly in the backbone to exert attention. The human visual perception process is used in [18] to obtain approximate information about the object position. It helps the model gradually focus on the correct object area, reducing the training cycle. Sparse R-CNN [19] replaces hundreds of thousands of candidates from an RPN network with a small group of suggestion boxes, and directly predicts the output, which avoids all the work related to candidate target design and many-to-one label allocation. DINO [20] improves upon previous DETR-like models in performance and efficiency by using a contrastive way for denoising training, a mixed query-selection method for anchor initialization, and a look-forward-twice scheme for box prediction.

DETR-like methods focus on attention at the macro level and constraint optimization for query embedding. However, they ignore the analysis of inefficient attention from the target’s perspective. There are two main challenges: (i) the sampling point of a target falls on other targets or background regions in encoding, and its attention is focused on non-identical targets, causing target attribute diffusion; or (ii) object queries are linked to too many background tokens, resulting in non-target interference in decoding.

To tackle target attribute diffusion and the non-target interference challenge of transformer-based object detection, we propose a target-aware token design for transformer-based object detection. The main contributions of this paper are summarized as follows.

- A target-aware sampling method is proposed to encourage the sampling patterns to converge inside the target region and obtain its representative encoded features. It consists of a set of multi-pattern parallel sampling strategies. More specifically, the small sampling pattern extracts the detailed features, the large sampling pattern obtains the larger receptive field features, and the vertical–horizontal sampling pattern approximates the target boundary. These are further fused together to strengthen the connection between the same targets and mitigate target attribute diffusion.
- A target-aware key-value matrix is proposed to be directly weighted on the feature map to reduce the interference of non-target regions. For that, we propose a learnable embedding matrix of the relationship between the target and the non-target to replace the original key-value matrix in DETR to calculate the self-attention weights.

The rest of this paper is organized as follows. The motivation for our proposed approach is presented in Section 2. Then, the proposed approach is described in Section 3 and evaluated in Section 4. Finally, Section 5 concludes this paper.

## 2. Motivation

In object detection, the attention mechanism uses a mask to describe the relationship between the target and the background, forming a new visual attention weight map. However, the mask convolution at the target boundary may spread the target attributes beyond the boundary and cause the inaccurate positioning of the target. In the calculation of self-attention weights, if the target area contains the weight of the background area, the target attribute is diffused.

The target attribute diffusion can be explained in the following two ways. Firstly, in self-attention, the feature vector *F* will generate three vectors *Q*, *K*, *V* through dimension reduction mapping, where *Q* is the query vector; *K* is the key vector, representing the matching standard of the feature vector; and *V* is the value vector, representing what the feature contains. The result of the inner product of *Q* and *K* represents the degree of similarity between the current feature and the standard matching value. The feature weighted by the result of *Q* and *K* matching on *V* is defined as self-attention, which includes the contribution of each position in the feature space to the current feature point. Self-attention is expressed as

(1) $$\mathrm{self}-\mathrm{attention}\left(K,Q,V\right)=Softmax\left(\frac{Q\cdot K^{T}}{\sqrt{d_{k}}}\right)V,$$

where Softmax() is the activation function, $d_{k}$ is the normalization coefficient, and the square root value $\sqrt{\left(d_{k}\right)}$ is used to reduce the sensitivity of attention to the feature dimension and make the gradient more stable.

Secondly, the sparse spatial sampling attention mechanism only calculates the weighted summation among a small set of sampling points, indicating the similarity of the point to the whole points set as

(2) $$\mathrm{sparse}-\mathrm{attention}=\sum_{k=1}^{K}A_{k}V_{p+\Delta p_{mn}},$$

where *K* is the number of points in the sampling range, $A_{k}$ is the attention weight at the sampling point *k*, and $\Delta p_{mn}$ is the two-dimensional offset relative to the reference point *p*. The idea of sparse attention draws on deformable convolution, which also generates offset and bilinear interpolation calculation features. The difference is that the feature of deformable convolution is obtained by multiplying the convolution kernel weight by the feature of the sampling position, and the feature of sparse attention is obtained by multiplying the normalized similarity coefficient of the current reference point and the sampling point by the feature of the sampling position. Sparse attention only computes the similarity of each point to a small set of sampled points. It also has the phenomenon of the diffusion of target attributes.

For the DETR-like structure, the calculation process from the feature-map token to the target can be expressed as

(3) $$T=\sum_{\left(i,j\right)}^{\left(H,W\right)}B_{ij}\sum_{\left(m,n\right)}^{\left(H,W\right)}A_{mn}\cdot t_{mn},$$

where *T* represents the target query that can obtain the category and border information of the object through linear mapping, (m,n) and (i,j) are the traversal points in the encoding and decoding stages (they will represent sampling points in sampling attention), $A_{mn}$ and $B_{ij}$ are the corresponding attention weights, and $t_{mn}$ is the feature map token. From the perspective of target awareness, the token-target process above can be written as

(4) $$T=\sum_{(i,j)\in BG}^{}B_{ij}\sum_{\left(m,n\right)}^{\left(H,W\right)}A_{mn}\cdot t_{mn}+\sum_{(i,j)\in FG}^{}B_{ij}\left(\sum_{(m,n)\in T}^{}A_{mn}\cdot t_{mn}+\sum_{(m,n)\notin T}^{}A_{mn}\cdot t_{mn}\right),$$

where BG is the background region, FG is the foreground region, and *T* is the region corresponding to the target *T*. For object detection, $\sum_{(i,j)\in BG}^{}B_{ij}\sum_{\left(m,n\right)}^{\left(H,W\right)}A_{mn}\cdot t_{mn}$ represents the background interference, $\sum_{(m,n)\notin T}^{}A_{mn}\cdot t_{mn}$ represents the target attribute diffusion, and only $\sum_{(i,j)\in FG}^{}B_{ij}\sum_{(m,n)\in T}^{}A_{mn}\cdot t_{mn}$ is the efficient information for object detection.

**Figure 1:**
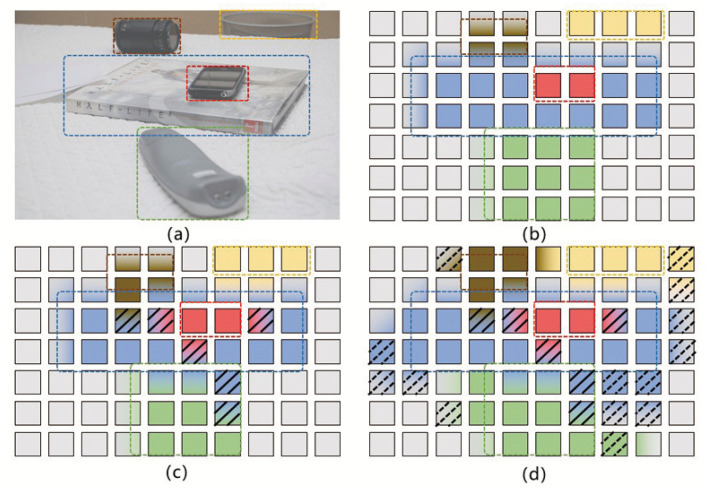
A conceptual overview of target attribute diffusion in attention-based object detection. (**a**) is the original image, (**b**) is the ideal attention-encoding feature map, (**c**) is the actual attention-encoding feature map with target attribute diffusion, (**d**) is the actual attention-encoding feature map with target attribute diffusion and background interference.

To illustrate these two phenomena, Figure 1a presents a test image containing multiple targets (such as camera lenses, mobile phones, etc.). Different targets are framed by dotted lines. We make the following observations concerning the feature map calculated from this test image.

- Firstly, before attention encoding, each point on the feature map is the feature extracted by the convolution kernel in a specific region of the original image, regardless of points outside the region. Each dotted box represents a specific object, all feature blocks are not related, and there is no target attribute diffusion phenomenon or background interference.
- Secondly, the feature map should have clear object information, as shown in Figure 1b; the feature block containing the target area should only be associated with other areas of the same object (each target has a different color, red means mobile phone, brown means camera lens). When decoding the object query, the position and bounding box of the object can be obtained according to the associated feature block. The feature blocks of the background area are not associated with the target area, carry no object information, and will not cause the diffusion of target attributes or background interference.
- Thirdly, due to the mechanism of attention weighting calculation, some sampling points of a certain target will fall in the region that does not belong to this target, this part of the sampling weight in (4) is expressed as $\sum_{(m,n)\notin T}^{}A_{mn}$. As shown in Figure 1c, the red region representing a mobile phone’s attribute diffuse to the blue region representing a book; the book’s attribute also diffuse to the lens region (brown) and the razor region (green). This is the phenomenon of target attribute diffusion (tokens highlighted with slashes in the figure). If the encoded feature token contains more attributes that do not belong to the same target, the target-aware ability will be reduced. It is difficult for object queries to establish effective associations with such weakly target-aware tokens, ultimately leading to false or missed detections.
- Fourthly, the background region also contains target information; this part of attention weight is expressed as $\sum_{(i,j)\in BG}^{}B_{ij}$. As shown in Figure 1d, the gray blocks are the background tokens, but some of them present different colors due to the doping target information (tokens highlighted with dotted lines in the figure). In the decoding process, object query will also confuse such tokens with target tokens, which affects the accuracy of detected object boundaries. This is the phenomenon of background interference.

The aforementioned observations of the target attribute diffusion challenge of transformer-based object detection motivate us to develop a new target-aware token design in the following section. Since $A_{mn}$ and $B_{ij}$ are the attention weights of each token, which satisfy the constraints of $\sum A_{mn}=1$ and $\sum B_{ij}=1$, we use *A* and *B* instead of $\sum_{(m,n)\in T}^{}A_{mn}$ and $\sum_{(i,j)\in BG}^{}B_{ij}$; (4) can be rewritten as

(5) $$T=\left(1-B\right)\left(A\sum_{(m,n)\in T}^{}t_{mn}+\left(1-A\right)\sum_{(m,n)\notin T}^{}t_{mn}\right)+B\sum_{\left(m,n\right)}^{\left(H,W\right)}t_{mn},$$

where $\sum_{(m,n)\in T}^{}t_{mn}$ is the feature token with high object perception; the higher the content of this part, the more accurate the detection result. We can improve the target-aware ability of the token by increasing the attention weight *A* and decreasing the attention weight *B*.

## 3. Method

**Figure 2:**
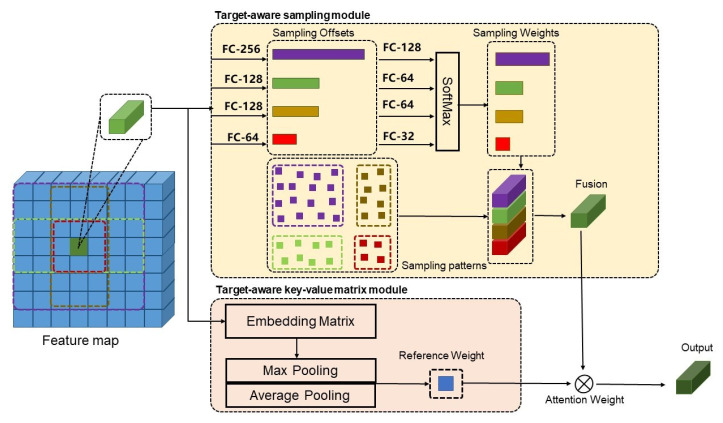
The overview of the proposed focal DETR with the target-aware token design. It consists of two key components: (i) a target-aware sampling module and (ii) a target-aware key-value matrix.

The overall structure of our proposed focal DETR is depicted in Figure 2. Our design consists of two key components: (i) a target-aware sampling module, which forces the sampling to converge inside the target region and obtain its representative encoded features and (ii) a target-aware key-value matrix, which is a unified learnable embedding matrix which is directly weighted on the feature map to reduce the interference of non-target regions.

### 3.1. Proposed Target-Aware Sampling

**Figure 3:**
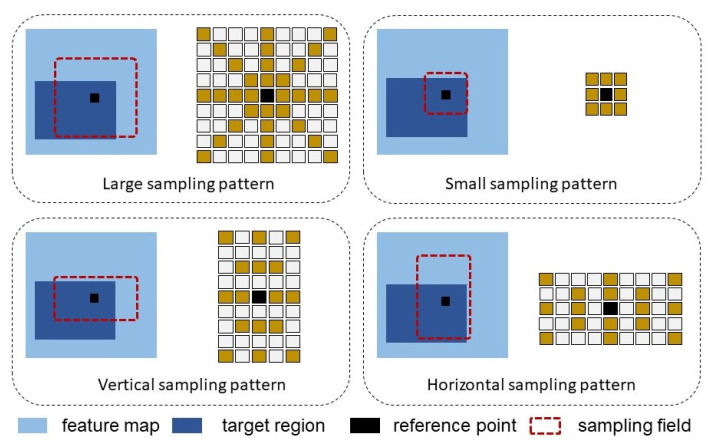
Proposed target-aware sampling patterns including a large sampling pattern, a small sampling pattern, and two rectangular orientation patterns.

We propose a composite-pattern sampling method to improve the attention ratio among sampling points by explicitly constraining the range of sampling points within the target region. It includes a small pattern, a large pattern, and two rectangular orientation patterns. The sampling position is defined as initialized position + offset, where the initial position is absolute location according to the sampling patterns, while the offset is learned by the FC layer. The following four sampling patterns are defined as shown in Figure 3.

- **Large sampling pattern.** The sampling side length is $2L_{0}$, which is consistent with the initial sampling layer. This is used to obtain larger receptive-field features and maintain the network’s grasp and perception of the integrity of the target.
- **Small sampling pattern.** The sampling side length is $\frac{L_{0}}{2}$. Compared with the large pattern with $2L_{0}$ as the sampling side length, using a smaller sampling side length means less misallocated attention. Focusing on features near the reference point makes it easier to converge inside the object, suppressing the attention weights imposed outside the target.
- **Vertical and horizontal sampling patterns.** This consists of two sampling layers, the length of the sampling frame is $L_{0}\times 2L_{0}$ and $2L_{0}\times L_{0}$, respectively, the region is $R_{\left|\right|}$ and $R_{=}$, which can adapt the contour of objects to arbitrary shapes. Our sampling strategy takes the scales and directions into account; for a person, the vertical mode is more relevant and for a vehicle frame, the horizontal mode is dominant.

**Figure 4:**
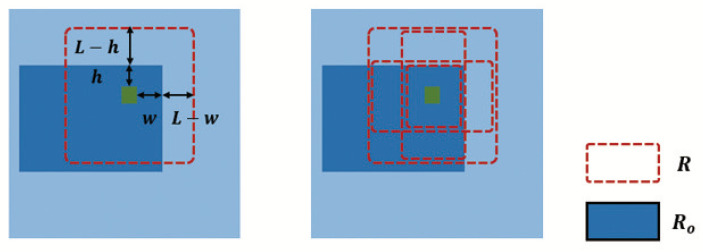
The sampling-pattern comparison. *R* represents the sampling area, $R_{o}$ represents the target area, *w* and *h*, respectively, represent the horizontal and vertical distances from the point $P_{s}\left(x,y\right)$ to the target boundary.

The large sampling pattern is the sampling pattern of Deformable DETR. The side length of the sampling frame is 2L. *R* represents the sampling area, $R_{o}$ represents the target area, and $R_{out}$ represents the sampling area outside the target, as shown in Figure 4. Suppose the reference point $P_{s}\left(x,y\right)$ is located at the target boundary, the sampling point set is S=|R|, and $S_{out}=\left|R-R_{o}\right|$ is the sampling point outside the target area. The misallocated attention is defined as the target-unrelated information quantity $I_{u}=\left(L-h\right)\left(2L+1\right)+\left(L-w\right)\left(2L+1\right)-\left(L-h\right)\left(L-w\right)=3L^{2}+\left(2-h-w\right)L-h-w-hw$, where *w* and *h*, respectively, represent the horizontal and vertical distances from the point $P_{s}\left(x,y\right)$ to the target boundary, which is independent of the value of *L* and is smaller than *L*. Since $L=\frac{\left(L+L\right)}{2}>\frac{h+w}{2}>\frac{h+w-2}{6}$ (*L* is always in the monotonically increasing interval), when *L* decreases, $I_{u}$ also decreases, but excessively decreasing the value of *L* will make the network focus on the details, and ignore the overall grasp and perception of the object. If the value of *L* is too large, the misallocated attention will increase, and the target attribute diffusion will be aggravated.

The sampling range of the composite pattern remains unchanged, the sampling point set is $S=\{s\in R+R_{s}+R_{=}+R_{\left|\right|}\}$, and the amount of target-independent information introduced by the small pattern sampling branch is $\frac{1}{4}L_{0}\left(\frac{3L_{0}}{4}-h-w+2\right)-h-w-hw$. The vertical–horizontal box pattern is $L_{0}\left(3L_{0}-\frac{5}{2}h-\frac{5}{2}w+3\right)-2h-2w-2hw$ and the increment in irrelevant information is smaller than the increment in the number of sampling points. That is to say, the proportion of target-aware attention in the feature token is increased, the increment is recorded as At and (4) can be rewritten as

(6) $$T=\left(1-B\right)\left(\left(A+A_{t}\right)\sum_{(m,n)\in T}^{}t_{mn}+\left(1-A-A_{t}\right)\sum_{(m,n)\notin T}^{}t_{mn}\right)+B\sum_{\left(m,n\right)}^{\left(H,W\right)}t_{mn}.$$

By constraining the location of the sampling points, the attention weight proportion of the same target is increased, and the target-aware ability of feature token is enhanced.

**Figure 5:**
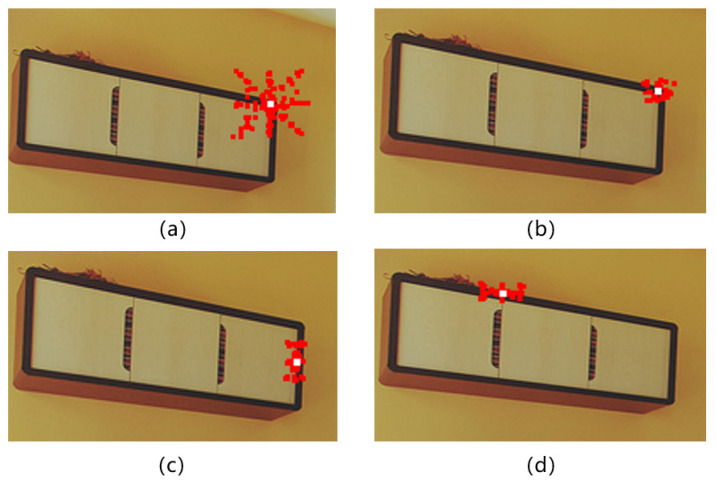
Visualization of sampling point locations after encoding. (**a**) is the large pattern, (**b**) is the small pattern, and (**c**,**d**) are the horizontal and vertical patterns, respectively. The white points are the reference points, and the red points are the sampling points.

To verify the proposed target-aware sampling, a test is conducted in Figure 5, which shows the location of sampling points after six-layer encoding. In this test image, the target is a cabinet, the white points are the reference points, and the red points are the sampling points. As seen in Figure 5, we make the following observations. Firstly, for boundary points, when the initial sampling side length is set to $2L_{0}$, after 6 layers of large-pattern encoding, some sampling points still fall outside the cabinet region. By adding the small mode, most of the sampling points are constrained in the cabinet area, and the attention weight outside the area is suppressed. By adding the vertical–horizontal box pattern in both directions, the sampling points converge to the vertical and horizontal boundaries of the cabinet. Secondly, for interior points, most of the sampling points are concentrated in the target, which is used to grasp the integrity of the object.

### 3.2. Proposed Target-Aware Key-Value Matrix

We propose a key-value degenerate target-aware embedding module. It uses a learnable embedding matrix to replace the key matrix generated from self-attention computation, reducing the interference effect of background tokens on decoding. The embedding matrix is randomly initialized and eventually mapped by the network.

In DETR, the *Q*-*K* inner product reflects the strength of the correlation among tokens. In theory, the accumulated sum of each token’s query to all tokens’ key in feature map can be regarded as the evaluation value of the target information. Generally, the background evaluation value is much smaller than that of the foreground. Taking it as the distribution map of the target information can reduce the interference of the background tokens. However, the implementation of this method will face the following two problems.

Firstly, the *Q*-*K* inner product sum is based on traversal self-attention, so the computational complexity and the number of tokens are in a square relationship. High-resolution feature maps are not suitable for self-attention (the number of inner products for a 200×200 feature map is 1,600,000,000, a general GPU cannot afford such a large calculation).

**Figure 6:**
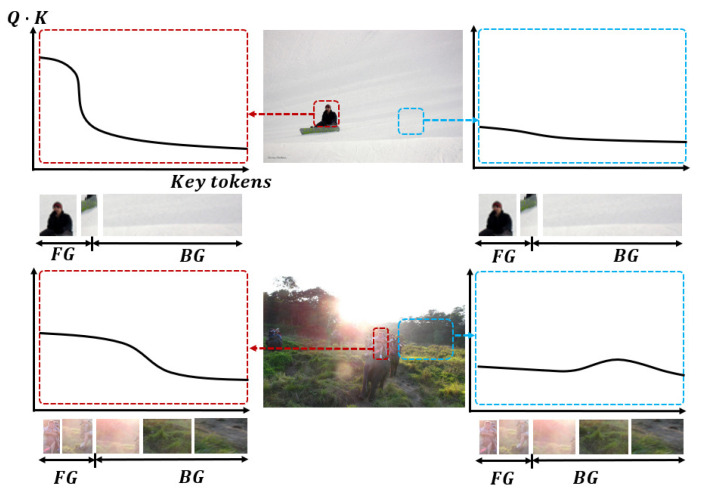
Distribution of *Q*–*K* inner product values of different pictures, The upper picture is a simple case: the object and the background have clear boundaries, the lower picture is a more complex situation: the object and the background are mixed together.

Secondly, the target information reflected by the *Q*-*K* inner product is not absolute and uniform; the distribution of the target information is more accurate only when the image object is sufficiently significant and the background is sufficiently monotonous. As shown in the upper picture in Figure 6, the boundary between the object and the background is clear, the self-attention of the foreground token is concentrated in the object region, and the distribution of the background token is scattered. The lower picture in Figure 6 shows a situation where the distribution is more complicated: the targets and the background are mixed together, and the distribution of the self-attention of the foreground and the background is similar. At this time, the interference caused by the background token cannot be eliminated. On the other hand, if a target consists of multiple structures with different textures (such as a person’s shirt and pants), the inner product sum of the target region will decrease. This relative relationship will affect the accuracy of object information distribution.

**Figure 7:**
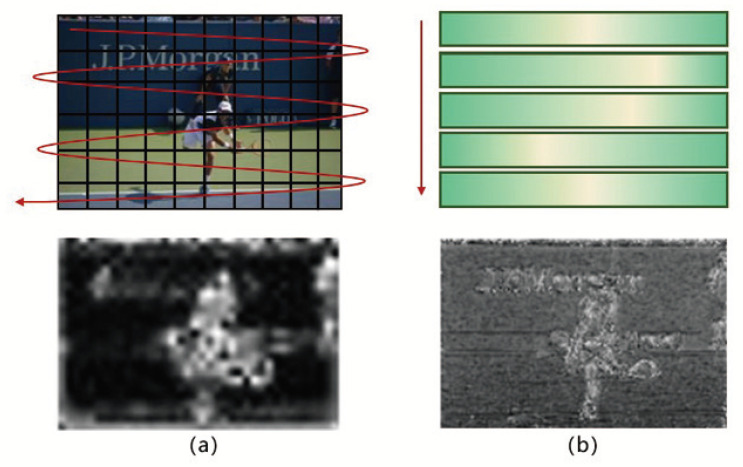
(**a**) is the accumulation of attention among reference points and (**b**) is between reference points and the matrix. (**b**) has less computation, rich details and sharp boundaries.

In view of this, we propose to use a unified, learnable embedding matrix *W* to replace the original query matrix *Q*. The matrix provides a set of standard queries, and the characteristic token key only needs to calculate the inner product with standard query (Figure 7). The sum of inner product can reflect the closeness of the current token and standard matrix. The calculation of the relative relationship between the reference points is converted into the absolute relationship between the reference point and the standard matrix, which provides a unified standard for the distribution of object information, and this information is obtained in a non-traversal way. The degenerated key-value matrix replacement can reduce the proportion of the weighted value of the background region in the overall reference point and achieve background suppression. The target information reflected by the *Q*–*K* inner product of a token in self-attention is

(7) $$\begin{array}{ccc} A_{ij} & = & f\left(\sum_{c}^{N}q_{c}\cdot k_{c}^{ij}\right), \end{array}$$

(8) $$\begin{array}{ccc} A & = & \sum_{\left(i,j\right)}^{\left(H,W\right)}A_{ij}, \end{array}$$

where *f* represents division by the normalization coefficient and activation operation, *N* is the dimension of the token, and (H,W) is the length and width of the feature map. It can be seen that the target information is merged through the order of dimension to space. Our proposed target-aware matrix learns the target information carrying amount *I* through space-to-dimensional fusion

(9) $$\begin{array}{ccc} I_{c} & = & f\left(\sum_{i}^{R}q_{i}\cdot k_{i}^{c}\right), \end{array}$$

(10) $$\begin{array}{ccc} I & = & pool(I_{c}), \end{array}$$

where *R* is a fixed number of standard reference points and pool represents the arithmetic mean of average pooling and max pooling. By using a key-value degenerate target-aware matrix, the relative attention between tokens and tokens is converted into standard attention between tokens and reference points.

This replacement method has two advantages. Firstly, the importance of the reference point itself is emphasized. Comparing the calculation of the relativity of the *Q*–*K* inner product, the degenerate embedding matrix *W* is used to fuse the object information of each dimension of the reference point, which can better reflect the distribution of object information in the image. All reference points are pooled and fused after the common matrix’s *W* mapping. Each reference point can be linked through *W* to measure the content of the target information. It is more effective to reduce the interference of background regions. Secondly, since there is no need to explicitly construct pairwise relationships for all reference points, the saved computing resources can use higher resolution feature maps, which are simpler and more accurate than self-attention calculations.

The target-aware embedding module adds a target attention weight $B_{t}$ (or $b_{n}$) to each token before decoding to reduce the interference of the background

(11) $$T=B_{t}\cdot \left(1-B\right)\left(\left(A+A_{t}\right)\sum_{(m,n)\in T}^{}t_{mn}+\left(1-A-A_{t}\right)\sum_{(m,n)\notin T}^{}t_{mn}\right)+B\cdot B_{n}\sum_{\left(m,n\right)}^{\left(H,W\right)}t_{mn}.$$

**Figure 8:**
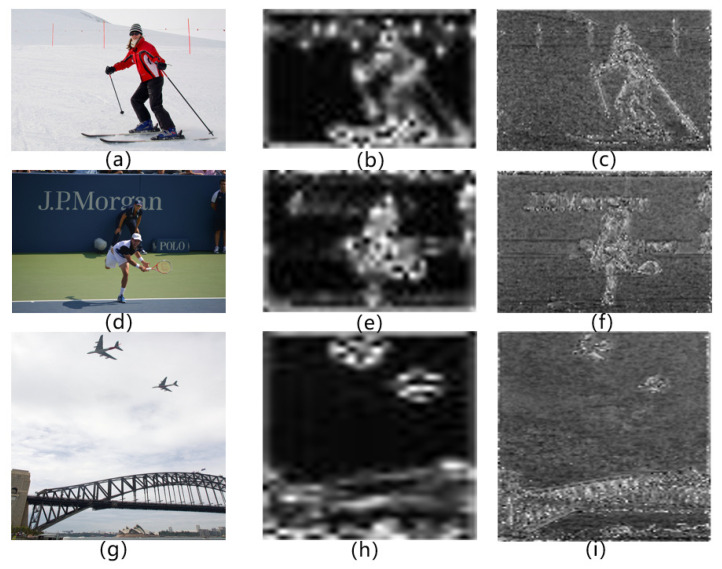
Visualization results of the test images (**a**,**d**,**g**) and their original self-attention layer (**b**,**e**,**h**) and the modified attention with our target-aware key-value matrix (**c**,**f**,**i**). The higher the brightness, the greater the attention weight.

Figure 8 illustrates the results obtained by the original self-attention layer after accumulating global attention at each point, and the visualization results of the weight branch. The higher the brightness, the greater the attention weight, that is, the more target information content. The attention is concentrated on the target areas such as people and buildings, which shows that the network does not need to explicitly construct the pairwise relationship for all points. Compared with the accumulation method of global attention in the middle column, the method on the right can provide a more detailed attention distribution map, the object offset is smaller, and the boundary position is sharper, which is more suitable for object-detection tasks. It is only necessary to learn object information according to the characteristics of each region itself, so as to judge the amount of target information carried in the region and apply appropriate attention weights. In this way, the proportion of $\sum_{p}^{p\in BG}V$ in the overall reference point is suppressed, thereby reducing the interference of the background region.

### 3.3. Proposed Focal DETR Structure

The overall structure of our focal DETR model follows the design of DETR. The CNN backbone is responsible for feature extraction. The extracted features are directly used as the input tokens of the encoder after being linearly mapped in equal dimensions. The encoder–decoder structure is responsible for establishing feature–feature and feature–object connections, and the final prediction module is responsible for outputting the final category and bounding box.

**Backbone.** We choose ResNet50 [21] to extract features of different levels. The feature maps of all levels are unified to 256 dimensions through a 1×1 convolution, respectively, and finally connected.

**Position embedding.** The position embedding [22,23] is divided into two parts. The first part uses trigonometric functions to encode the parity dimension of each position for the spatial position on the feature map. The absolute position is first encoded by row and column, and, then, sine and cosine encoding are applied to obtain $PE\left(pos,2i\right)=sin\left(\frac{pos}{10000^{\frac{2i}{d}}}\right)$ and $PE\left(pos,2i+1\right)=cos\left(\frac{pos}{10000^{\frac{2i}{d}}}\right)$, where pos is the actual position of the feature, and *d* is half the number of feature’s dimension. Finally, the encodings of rows and columns are concatenated together.

**Focal DETR encoder.** We use a composite-pattern sampling module to replace the sampling attention module in deformable DETR [12]. Based on the original sampling layer, we add three parallel sampling layers. The original sampling layer is a large pattern, the number of sampling points is *N*, the number of newly added small-pattern sampling points is N/4, and the number of vertical and horizontal box-pattern sampling points is N/2. Each pattern contains a sampling offset branch and a sampling weight branch. In addition, a feature fusion module is added to summarize the features of the four patterns. We will present experimental results to justify the choice of the fusion strategy.

**Focal DETR decoder.** We insert a learnable embedding matrix to linearly map the feature map before the cross-attention layer of the deformable DETR [12], and fuse the information across all dimensions through the pooling layer to obtain the target attention weight of every point in the feature map. The feature map weighted by the target attention is used to calculate cross attention with object query. The self-attention layer in the decoder is the same as that used in both DETR [11] and deformable DETR [12].

In the self-attention decoding process, the object query needs to traverse all the feature tokens to find the stuff or thing category information contained in them. The stuff type is widely distributed and has a greater association with other objects, while the thing type is concentrated and has a greater association within the same object. Due to the characteristics of traversal, with self-attention decoding it is hard to extract the information in the distribution concentration of the thing type, causing a large amount of irrelevant information interference and affecting the detection effect. In addition, due to the use of multi-scale feature maps, self-attention will bring excessive computational burden.

The sampling attention selects some sample points for attention calculation based on reference points and learned biases. In this way, it is difficult to extract objects of the stuff type. First, the relevant information of the reference point is learned from the network. For an item such as a car, the reference point can be set at its center, which has a clear physical meaning. However, for the amorphous area, it is difficult to find a reference point with a clear meaning to replace this area, so the learning difficulty of the reference point is higher. Second, since the background area generally overlaps with multiple objects, and its own features are not obvious, using a small-scale sampling attention, the object query will be attracted by the foreground targets with more obvious features. As a result, the important information of the stuff class is ignored, resulting in missed detection.

**Figure 9:**
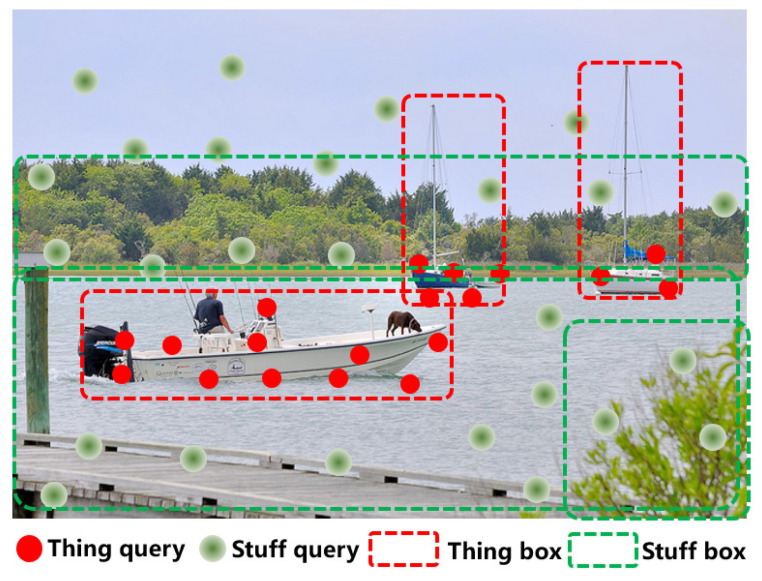
The stuff and thing query; self-attention is used to extract the centralized things, sampling attention is used to collect the scattered stuff.

In view of this, we deal with the stuff and thing classes separately. We use global self-attention decoding to collect scattered and smooth stuff-class information, and use sampling attention to detect the more concentrated thing-class information (see Figure 9).

## 4. Experimental Results

In this section, we conduct four experiments to evaluate the performance of the proposed approach. Firstly, we compare the proposed approach with classical detection algorithms and variants of DETR [11]. Secondly, to study the effects of various sampling-point initialization patterns on the object-detection results, we compare the detection performance of the four sampling patterns. Thirdly, we compare the fusion effects of the sampling branches, including adding directly, feature splicing, and pooling, then select the optimal solution. Fourthly, an ablation study is carried out to analyze the improvement in the detection effect brought about by the target-aware sampling and target-aware key-value matrix, respectively.

### 4.1. Datasets and Metrics

Experiments were performed on the challenging MS COCO [24,25] benchmark dataset. The model was trained on COCO train2017 (11.8k) and evaluated on val2017 (5k). The evaluation indices include different *intersection and union ratio* (IOU) values and *average precision* (AP) under large, medium, small objects, respectively.

### 4.2. Implementation Details

We used ResNet-50 pre-trained on ImageNet as the feature-extraction network. The number of heads of each multi-head attention layer was set to 8; the sampling branch was 4; and the total sampling points of each branch were 128, 64, 64, 32, respectively; the number of layers for both encoder and decoder was 6, and the number of object queries was 300. We trained the model using the AdamW optimizer [26] with an initial learning rate of $10^{-4}$ (in particular, the feature-extraction network backbone has an initial learning rate of $10^{-5}$). The rest of the hyperparameters used the default values (i.e., $\beta_{1}=0.9$, $\beta_{2}$ = 0.999, weight delay $=10^{-4}$), the learning rate decays to 10% of the original every 40 epochs, and the batch size =1 for each batch of training. The proposed approach was implemented using PyTorch and developed using Nvidia GTX 2080Ti GPU.

### 4.3. Comparison with Other Object-Detection Methods

The comparison results with the classical object-detection algorithms and some variants of DETR are shown in Table 1. As seen in this table, our approach outperforms the DETR model, where the average AP is increased by 2.7, and the small-object detection is improved by 6.5. In addition, our approach outperforms the deformable-DETR by increasing the average AP by 0.9.

### 4.4. Evaluation of Various Sampling Patterns

**Figure 10:**
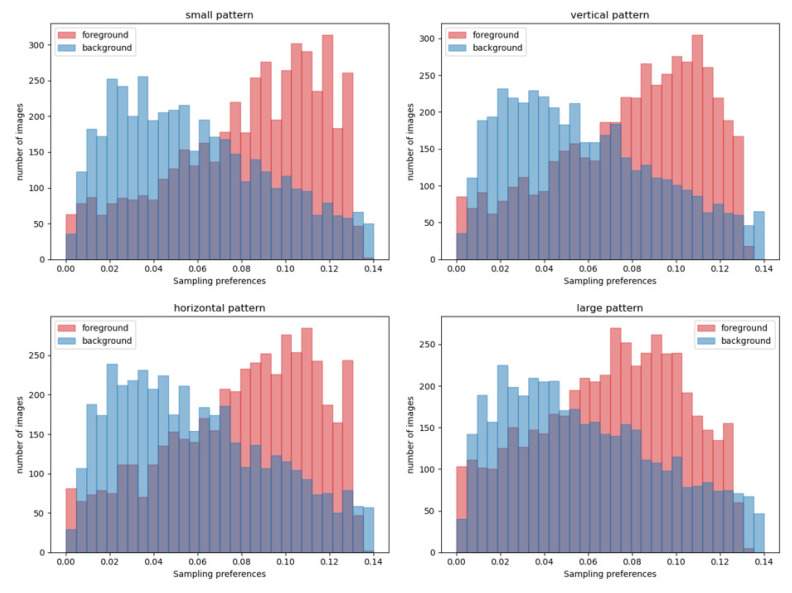
Statistical results of four sampling patterns. The abscissa represents the average sampling preference (red is the target preference, blue is the background preference), and the ordinate represents the number of pictures.

To study the positions of sampling points in each pattern, we recorded the offset coordinates of all sampling points belonging to the target tokens on the feature map, and calculated the final sampling positions. The number of sampling positions in the target region and background region were counted separately, and divided by the total number of corresponding tokens on the feature map as their sampling preference. The results of the four sampling branches on the coco test set are shown in Figure 10. The abscissa represents the preference of each image for the sampling region (red is the target preference, blue is the background preference), and the ordinate represents the number of images. All patterns are more inclined to samples in the target region, among which the small pattern is more obvious than the large pattern, and the vertical and horizontal patterns are similar.

**Figure 11:**
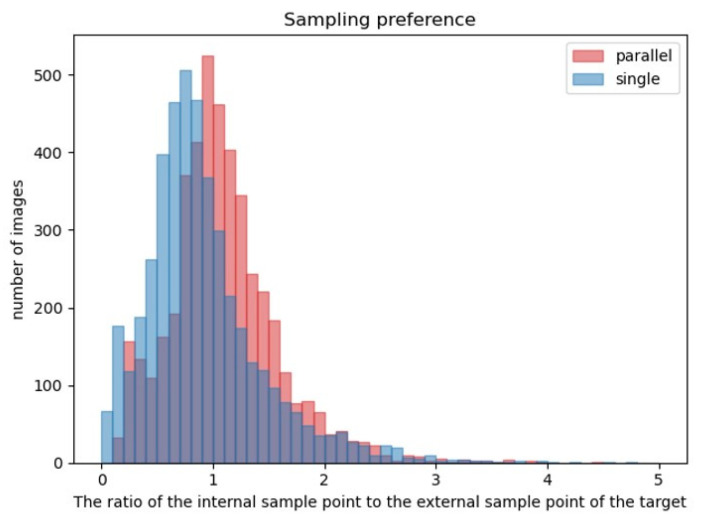
The visualization experiment of sampling points. The blue represents the Deformable DETR sampling strategy, and the red represents our strategy.

In addition, we conducted a visualization experiment of sampling points, and counted the ratio of internal sampling points to external sampling points of all objects in each picture. The results are shown in Figure 11. The blue represents the Deformable DETR sampling strategy, and the red represents our strategy. The proportion of sampling points using the parallel sampling strategy falling on the object is higher than that of unused ones.

To investigate the effect of multiple-sampling-point initialization modes on the object-detection results, four patterns were compared and their performances are shown in Table 2. In a single pattern, the detection result decreases by 1 average AP compared with that of multi-patterns, which proves that multi-patterns can improve the detection effect. In addition, the larger range pattern performs better than the smaller ones; the average AP of small targets in 3×3 mode is 0.6 lower than that of 9×9 mode, and the average AP of large targets is 1.9 lower. This also proves that if the sampling range is too small, that will make the network focus on the details, ignoring the grasp and perception of the wholeness of the object, and, consequently, reducing the detection quality.

### 4.5. Evaluation of Various Fusion Methods

There are three ways to fuse the four branch features from four samplings, including *adding directly*, *feature splicing*, and *pooling*. Table 3 compares the results using these three fusion strategies. Feature splicing splices the features $F\in R^{1\times 256}$ from the four branches into $F^{{}^{\prime }}\in R^{1\times 1024}$ by channel, and then reduces the dimension to $F^{\prime \prime }\in R^{1\times 256}$. The pooling is spliced into $F^{{}^{\prime }}\in R^{4\times 256}$ by pattern and then passes through the maximum pooling layer to obtain $F^{{}^{\prime \prime }}\in R^{1\times 256}$. Compared with direct addition, feature splicing and pooling reduce the average AP by 2.5 and 1.5, respectively. This is due to the fact that the features of the four branches are all sampled on the same value matrix. These two methods will map the features to a new space, breaking this consistency and increasing the difficulty of learning.

### 4.6. Evaluation of Target-Aware Matrix

**Figure 12:**
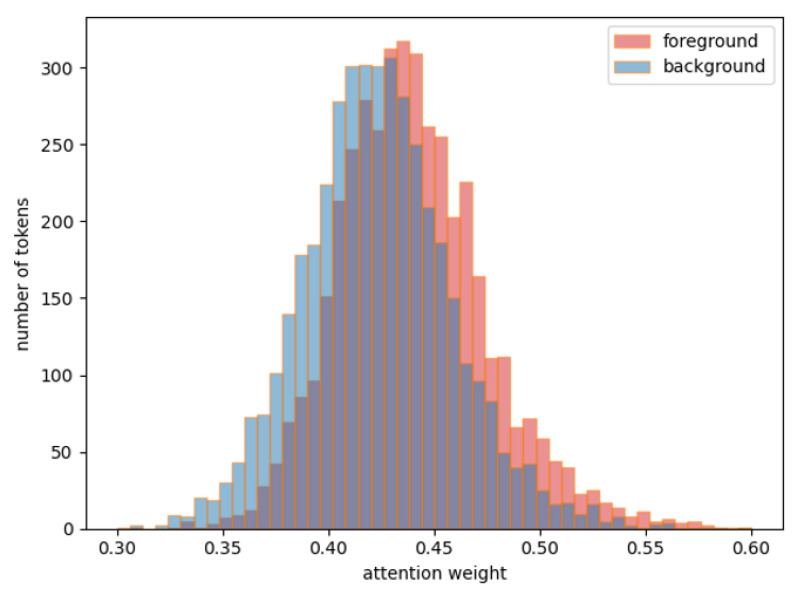
Attention weight of target-aware matrix; red is the target tokens, blue is the background tokens. The abscissa represents the average attention weight, and the ordinate represents the number of pictures.

To study the effect of the target-aware embedding matrix, we extracted all the tokens from the coco test set image through the third stage of the backbone, and divided them into foreground and background according to the position of the label. The embedding module calculates the attention weights of all tokens separately, and obtains the average-attention-weight distribution map (Figure 12) of the foreground and background tokens of each picture. The red part is the attention weight of the target tokens, and the blue part is the attention weight of the background tokens. The abscissa represents the average attention weight, and the ordinate represents the number of pictures. Overall, the attention weight of the target token is about 0.05 higher than that of the background part, which means that the target information carried by the feature token can be obtained using linear mapping and pooling fusion, and no explicit loss function constraints are required. This proves that adding a learnable embedding matrix and increasing the attention weight of the target part can effectively alleviate the interference of the background region.

### 4.7. Ablation Study

The ablation study was carried out to analyze the improvement in object detection by gradually adding our proposed two components. As seen in Table 4, after adding the target-aware sampling and the target-aware key-value matrix, the results improve by 1.0 *AP* and 0.9 AP, respectively. The constrained sampling in a compound pattern has obvious improvement in the detection effect of small and large targets (0.7 *AP* and 1.4 *AP*, respectively). Medium-sized targets show insignificant improvements (∼0.8%). The reason for this is that the introduced small-pattern sampling branch pays more attention to details, the encoding effect of small targets is more ideal, and the compound pattern makes it easier to constrain the sampling points inside the target when the target is large. In addition, with the help of the target-aware key-value matrix, it is easier for the network to capture small-sized objects that may be mixed in the background. Therefore, the detection effect of small targets is significantly improved compared to other-size targets (the improvement is 4.5% for small objects, and 1.8% and 1.3% for others).

## 5. Conclusions

A focal DETR was proposed in this paper with the development of a new target-aware token design. It effectively utilizes the target-aware sampling and a target-aware key-value matrix to effectively achieve higher object-detection performance, as verified on the MS COCO benchmark dataset.

## Figures and Tables

**Table 1:** The object-detection performance comparison on COCO val2017. The best performance is highlighted in bold format.

Model	AP	${\mathrm{AP}}_{50}$	${\mathrm{AP}}_{75}$	${\mathrm{AP}}_{S}$	${\mathrm{AP}}_{M}$	${\mathrm{AP}}_{L}$
Faster-RCNN [3]	42.0	62.1	45.5	26.6	45.4	53.4
RetinaNet [10]	40.8	61.1	44.1	24.1	44.2	51.2
FCOS [27]	41.0	59.8	44.1	26.2	44.6	52.2
TSP-FCOS [28]	43.1	62.3	47.0	26.6	46.8	55.9
TSP-RCNN [28]	43.8	63.3	48.3	**28.6**	46.9	55.7
DETR [11]	42.0	62.4	44.2	20.5	45.8	61.1
UP-DETR [29]	42.8	63.0	45.3	20.8	47.1	**61.7**
Anchor-DETR [15]	42.1	63.1	44.9	22.3	46.2	60.0
Conditional-DETR [14]	43.0	**64.0**	45.7	22.7	46.7	61.5
Deformable-DETR [12]	43.8	62.6	47.7	26.4	47.1	58.0
Ours	**44.7**	63.9	**48.5**	27.0	**47.5**	59.1

**Table 2:** The comparison of our focal DETR with various sampling-pattern methods on COCO val2017.

Pattern	AP	${\mathrm{AP}}_{50}$	${\mathrm{AP}}_{75}$	${\mathrm{AP}}_{S}$	${\mathrm{AP}}_{M}$	${\mathrm{AP}}_{L}$
Small	42.9	62.4	46.9	25.7	46.5	55.8
Rectangle-L	43.6	63.1	47.7	26.2	46.7	57.4
Rectangle-W	43.4	63.1	47.2	26.0	46.8	57.3
Large	43.8	63.3	47.5	26.3	47.1	57.7

**Table 3:** The comparison of our focal DETR with different fusion strategies on COCO val2017.

Fusion Method	AP	${\mathrm{AP}}_{50}$	${\mathrm{AP}}_{75}$	${\mathrm{AP}}_{S}$	${\mathrm{AP}}_{M}$	${\mathrm{AP}}_{L}$
Adding	44.7	63.9	48.5	27.0	47.5	59.1
Splicing	42.2	62.0	46.0	25.8	45.7	55.5
Pooling	43.2	62.7	47.1	25.8	46.2	57.3

**Table 4:** Ablation study.

Target-Aware Sampling	Target-Aware Key-Value Matrix	AP	${\mathrm{AP}}_{50}$	${\mathrm{AP}}_{75}$	${\mathrm{AP}}_{S}$	${\mathrm{AP}}_{M}$	${\mathrm{AP}}_{L}$
-	-	43.4	62.6	47.5	26.3	46.9	57.9
*√*	-	43.7	63.2	47.3	25.8	46.6	58.3
-	*√*	43.8	63.3	47.5	26.3	47.1	57.7
*√*	*√*	44.7	63.9	48.5	27.0	47.5	59.1

## Data Availability

Not applicable.
